# Endophytic Species of the Genus *Colletotrichum* as a Source of Bioactive Metabolites: A Review of Their Biotechnological Potential

**DOI:** 10.3390/microorganisms13081826

**Published:** 2025-08-05

**Authors:** Manuela Vitoria Nascimento da Silva, Andrei da Silva Alexandre, Cecilia Veronica Nunez

**Affiliations:** 1 Bioprospecting and Biotechnology Laboratory, Technology and Innovation Coordination, National Institute of Amazonian Research, Manaus 69067-375, AM, Brazil; mvnascimento99@gmail.com (M.V.N.d.S.); dreialexandre@gmail.com (A.d.S.A.); 2 Graduate Program in Biotechnology and Natural Resources of the Amazon, School of Health Sciences, Amazonas State University (UEA), Manaus 69050-010, AM, Brazil

**Keywords:** *Colletotrichum*, endophytes, secondary metabolites, biological activity, biotechnology

## Abstract

The genus *Colletotrichum* is widely known for its phytopathological significance, especially as the causative agent of anthracnose in diverse agricultural crops. However, recent studies have unveiled its ecological versatility and biotechnological potential, particularly among endophytic species. These fungi, which asymptomatically colonize plant tissues, stand out as high-yielding producers of bioactive secondary metabolites. Given their scientific and economic relevance, this review critically examines endophytic *Colletotrichum* species, focusing on the chemical diversity and biological activities of the metabolites they produce, including antibacterial, antifungal, and cytotoxic activity against cancer cells, and antioxidant properties. This integrative review was conducted through a structured search of scientific databases, from which 39 relevant studies were selected, highlighting the chemical and functional diversity of these compounds. The analyzed literature emphasizes their potential applications in pharmaceutical, agricultural, and industrial sectors. Collectively, these findings reinforce the promising biotechnological potential of *Colletotrichum* endophytes not only as sources of bioactive metabolites but also as agents involved in ecological regulation, plant health promotion, and sustainable production systems.

## 1. Introduction

Fungal endophytes are increasingly recognized for their abilities to produce a wide range of secondary metabolites with ecological and biotechnological significance. Among them, the genus *Colletotrichum* has gained attention due to its dual roles as a phytopathogen and as a symbiotic endophyte capable of synthesizing bioactive compounds, including polyketides, terpenoids, alkaloids, and sterols like ergosterol and *β*-sitosterol [1].

Fungal endophytes were first described by August Carl Joseph Corda in 1831 and formally published in 1837. *Colletotrichum* is ranked among the top ten most agriculturally and ecologically significant phytopathogenic fungal genera [2,3]. These genera belong to the phylum Ascomycota, class Sordariomycetes, order Glomerellales, and family Glomerellaceae, with the sexual morph named as *Glomerella cingulata* [4,5,6]. They are globally distributed and exhibit a striking diversity of lifestyles, including necrotrophic, hemibiotrophic, endophytic, latent, and saprophytic forms [2,7,8,9].

*Colletotrichum* species are widely known as etiological agents of anthracnose—a disease producing sunken necrotic lesions on leaves, stems, flowers, fruits, and roots [7,10,11,12]. This disease may occur both pre- and post-harvest, with especially damaging effects during fruit production. The economic burden of *Colletotrichum* infections is substantial, with losses reported across tropical, subtropical, and temperate crops, including cereals, legumes, fruit trees, vegetables, ornamentals, and grasses [7,10,13,14,15].

The genus was estimated to comprise hundreds of species associated with more than 2200 plant hosts. Some, like *C. orbiculare*, are highly host-specific (infecting cucurbits), whereas others, such as *C. gloeosporioides*, are generalists infecting over 470 hosts. Their geographical distribution varies: *C. kahawae* is regionally restricted, while *C. acutatum* and *C. simmondsii* are more common in Oceania, and *C. godetiae* predominates in Europe [16,17].

Although predominantly recognized as pathogens, many *Colletotrichum* species have been isolated as symptomless endophytes, especially in tropical regions. This dual behavior underscores the ecological complexity of the genus, as some species act as destructive pathogens, while others confer benefits to their host plants. Such functional plasticity presents both opportunities and challenges for biotechnological exploitation. Recent advances in omics-based tools, particularly genomics and metabolomics, have proven essential for deciphering the genetic and metabolic basis of this variability, enabling a more accurate discrimination between pathogenic and beneficial strains. Accurate strain selection is crucial for distinguishing beneficial from pathogenic lifestyles and preventing harm to non-target organisms or ecosystems [9,18,19,20,21].

Their ecological plasticities allow the same fungal isolate to behave as a mutualist or a parasite depending on host genotype and environmental conditions [22,23]. Under certain conditions, these fungi may promote plant growth, enhance drought tolerance, and increase resistance to biotic and abiotic stresses [24,25,26]. These benefits are often mediated by the production of indole-3-acetic acid (IAA), antifungal metabolites, and lytic enzymes and the induction of systemic resistance, supporting their role as effective biocontrol agents. Altogether, these attributes highlight the potential of endophytic *Colletotrichum* as sustainable alternatives to synthetic pesticides and fertilizers [21,27].

Among the most promising traits of endophytic *Colletotrichum* species is the production of bioactive secondary metabolites. Numerous studies have reported antifungal, antibacterial, phytotoxic, and cytotoxic compounds, highlighting their biotechnological values [13,20,28]. Notable compounds include colletotrichins A–C, colletotric acid, bisabolanoic acid A, and coletonic acid, with reported activities against pathogens like *Staphylococcus aureus*, *Klebsiella pneumoniae*, and *Cladosporium* spp. [29,30,31]. Additionally, the *C. dematium* isolate has shown activity against *Shigella flexneri*, *S*. *boydii*, *Salmonella enteritidis*, *S*. *paratyphi*, and *Pseudomonas aeruginosa* [25].

Beyond their pharmacological relevance, *Colletotrichum* endophytes also play important ecological and biotechnological roles by protecting host plants from pathogens, producing bioactive compounds, and mediating trophic interactions in ecosystems [32,33,34]. Despite their known roles and presence in tropical regions, research on endophytic fungi has focused mainly on temperate zones, leading to a geographical sampling bias. This limits our understanding of the true taxonomic and metabolic diversity of *Colletotrichum*, especially in biodiversity-rich tropical ecosystems that remain underexplored despite their potential to harbor novel metabolically diverse strains [21,35,36,37].

In this context, *Colletotrichum* emerges not only as a phytopathogen but also as a model organism for understanding endophytic fungal biology and plant–microbe interactions [20,38,39]. This review focuses on bioactive secondary metabolites produced by endophytic *Colletotrichum* species, and it is based on 39 selected studies. It highlights their chemical diversities and biosynthetic potential while discussing ecological relevance and prospective applications in agriculture, medicine, and the environment. By compiling evidence from diverse sources, including scientific articles, theses, and dissertations, this work also addresses current limitations and explores future directions to unlock the biotechnological potential of this genus.

## 2. Materials and Methods

This integrative review aimed to compile and synthesize current knowledge on bioactive secondary metabolites produced by endophytic *Colletotrichum* species. The strategy was designed to include a broad spectrum of sources relevant to chemical and biotechnological aspects. Literature research was conducted using four scientific databases: Google Scholar, ScienceDirect, PubMed, and SciFinder.

The search terms included combinations of “*Colletotrichum*”, “endophytic fungi”, “secondary metabolites”, and “bioactive compounds”. No date limits were imposed, allowing the inclusion of both historical and recent studies. Only full-text documents written in English or Portuguese were considered.

The inclusion criteria comprised original research, dissertations, and theses that reported the isolation or identification of biologically active secondary metabolites from *Colletotrichum* strains with endophytic behavior. These non-peer-reviewed sources were included to provide coverage of relevant data from underexplored regions and to compensate for gaps in the formal literature, particularly in tropical ecosystems. The exclusion criteria involved studies focused solely on the phytopathogenic role of *Colletotrichum* without reference to its endophytic activity or metabolite production, as well as duplicate records, restricted-access documents, or those unrelated to the research focus.

**Figure 1:**
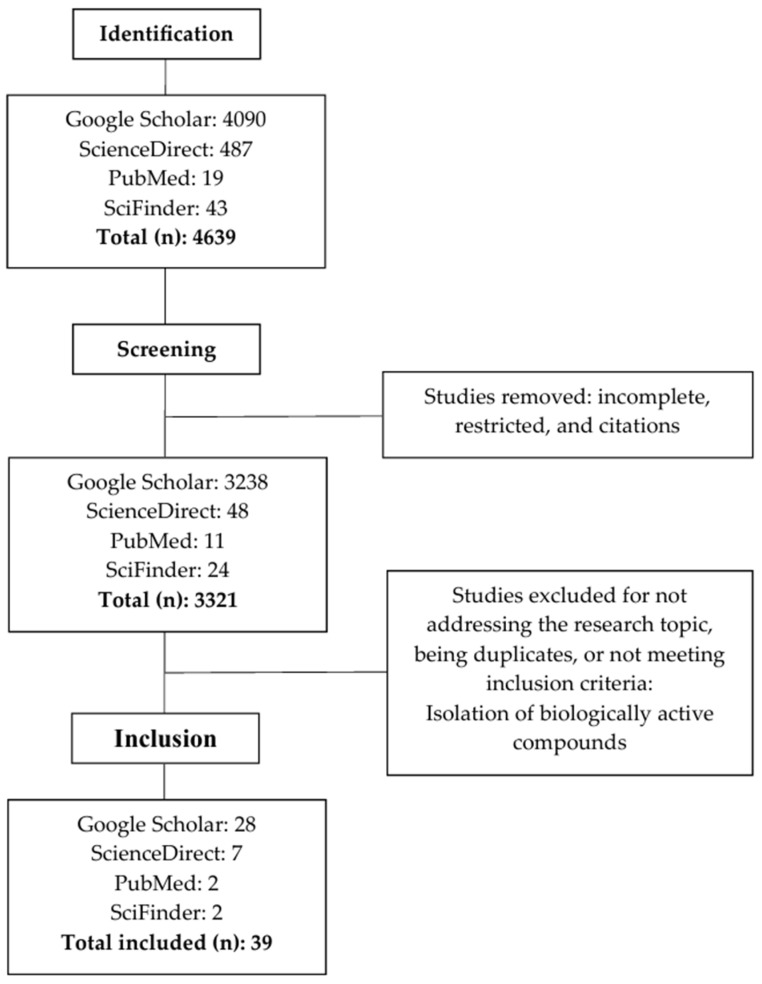
Flowchart of the article selection process.

From an initial pool of 4639 records, 3321 remained after removing duplicates and incomplete or restricted-access materials. All studies were screened manually by the author, and this was carried out based on individual reading of titles, abstracts, and full texts. No automation tools or software were used. Ultimately, 39 documents met all inclusion criteria and were selected for full analysis. The selection process is summarized in Figure 1.

The selected studies were organized according to metabolite class, biological activities, and fungal species involved. This manual categorization allowed the identification of patterns and knowledge gaps in the bioprospecting of endophytic *Colletotrichum* species.

## 3. Results

A total of 10 unidentified isolates and 13 distinct species of *Colletotrichum* were reported in biological and chemical studies. These included *Colletotrichum* sp., *C. fragariae*, *C. gloeosporioides*, *C. acutatum*, *C. capsici*, *C. dematium*, *C. siamense*, *C. taiwanense*, *C. fructicola*, *C. tropicicola*, *C. alatae*, *C. crassipes*, *C. brevisporum*, and *C. queenslandicum*.

**Figure 2:**
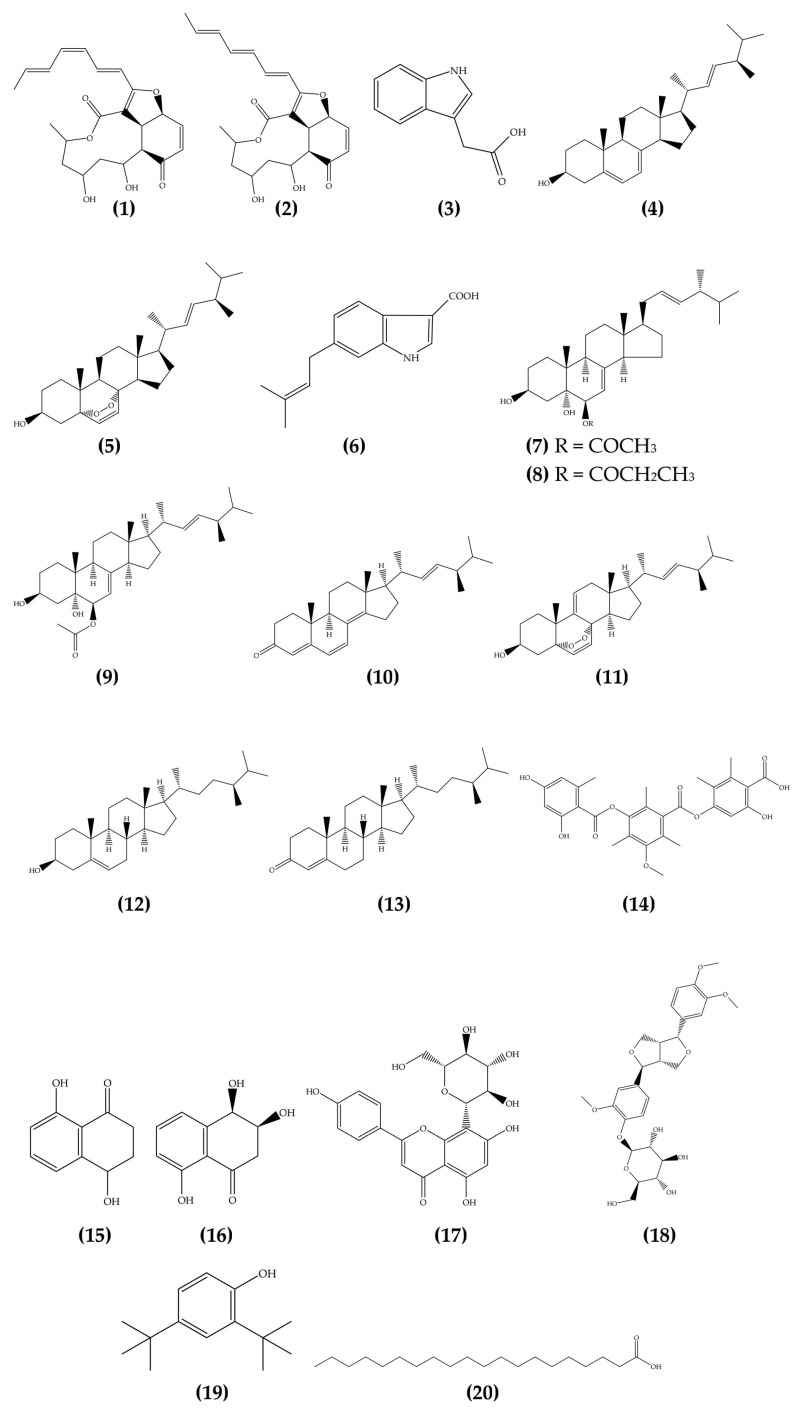

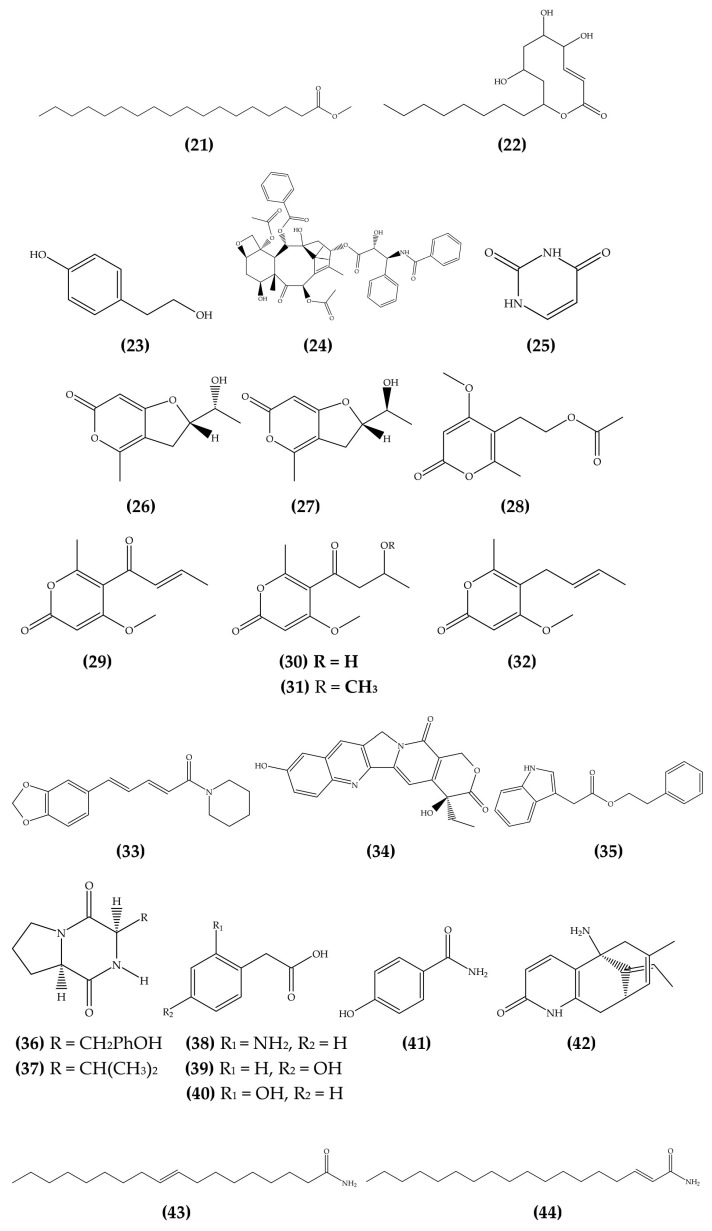

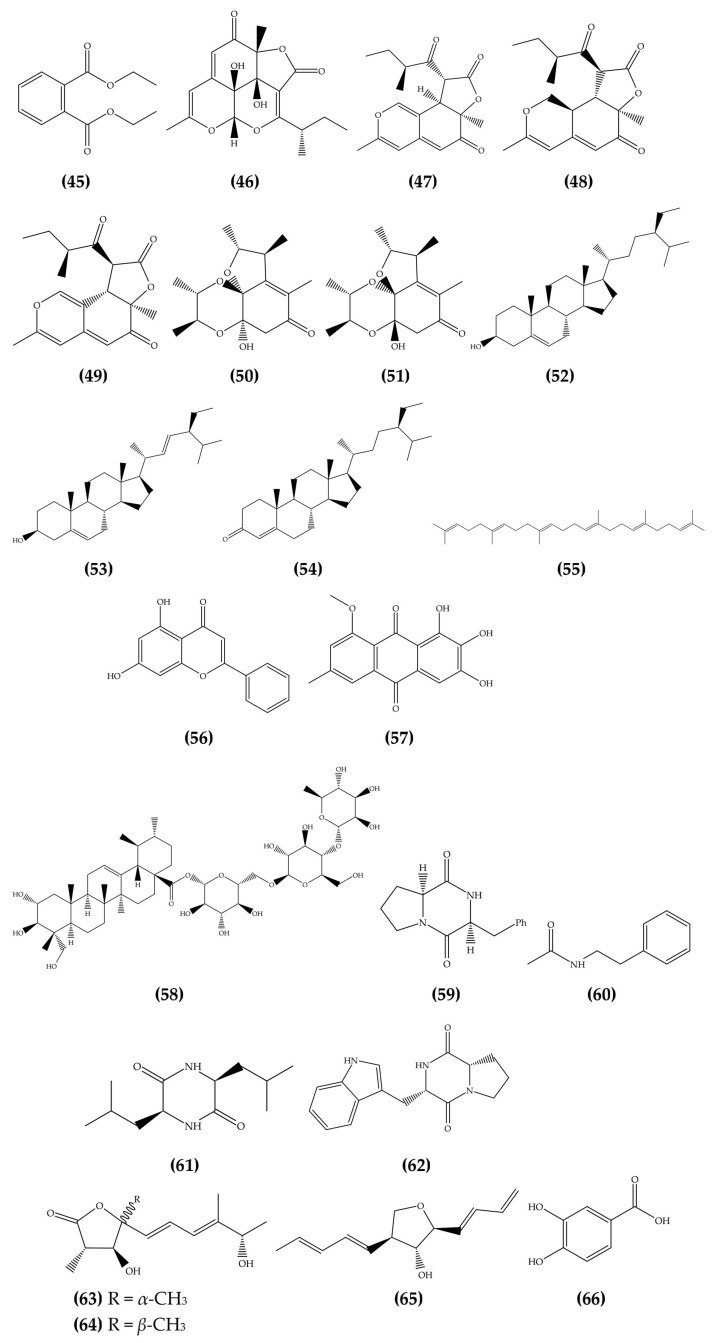

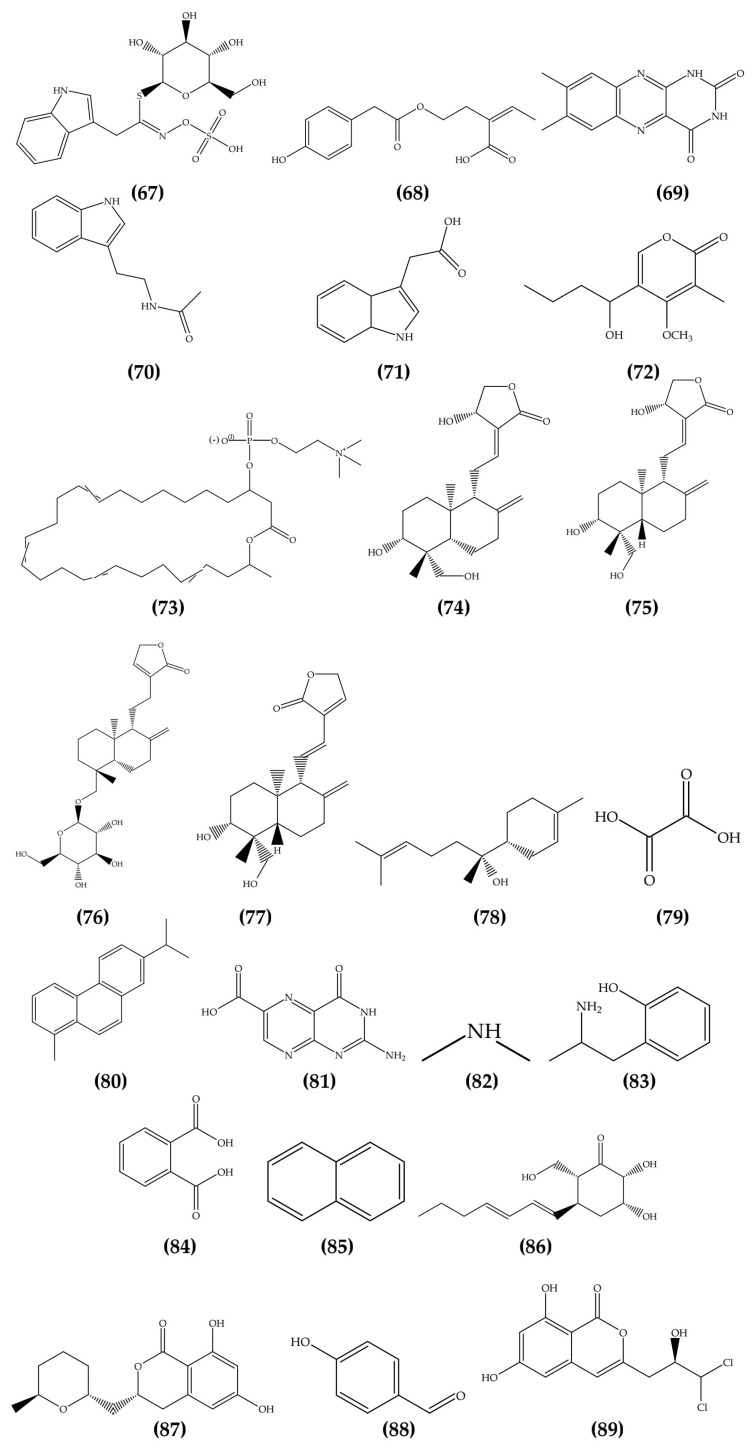

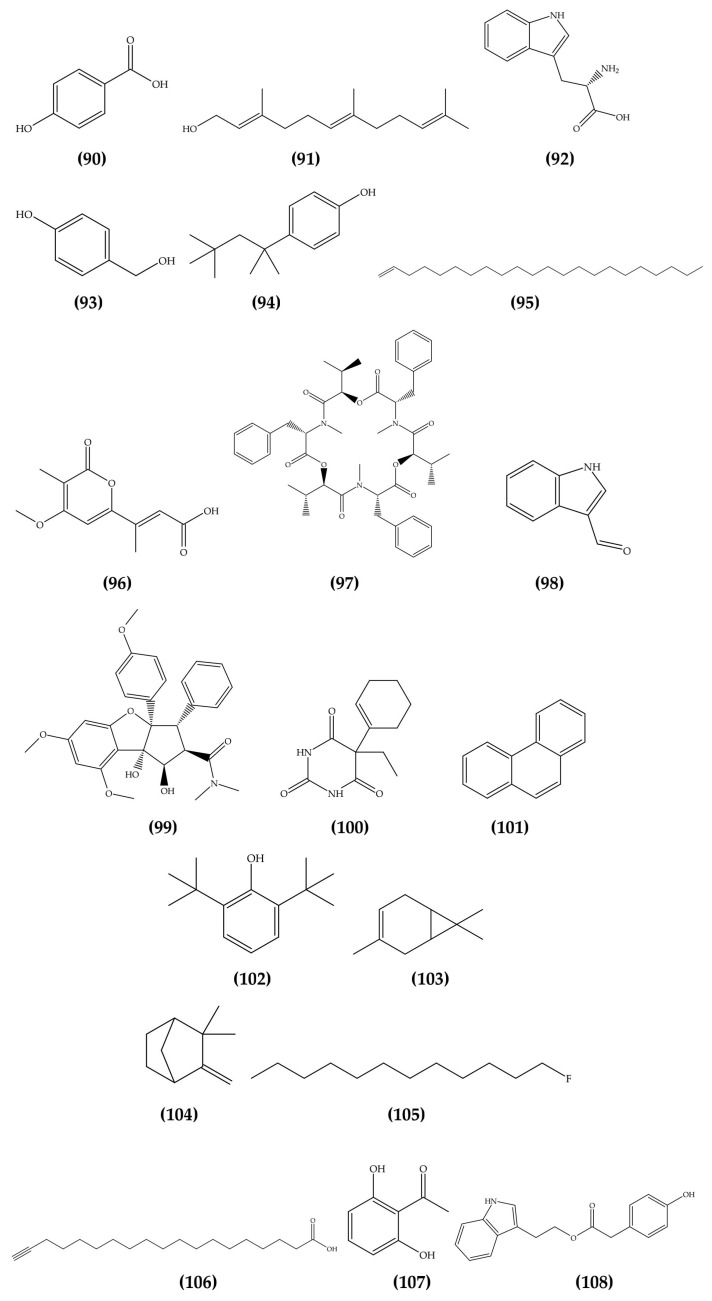

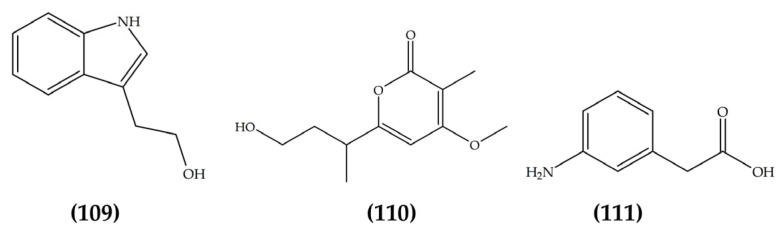
Chemical structures of bioactive compounds identified from endophytic *Colletotrichum* species.

These species were found to produce a wide range of bioactive compounds, including some also known to be synthesized by their host plants. The metabolites belong to diverse chemical classes and exhibit various biological activities, such as antimicrobial, cytotoxic, and antioxidant effects. Table 1 summarizes the endophytic species, host plants, identified metabolites, chemical classes, and associated biological activities. The chemical structures of selected compounds are presented in Figure 2. For clarity and historical context, the references in Table 1 are organized chronologically from older to more recent studies.

**Figure 3:**
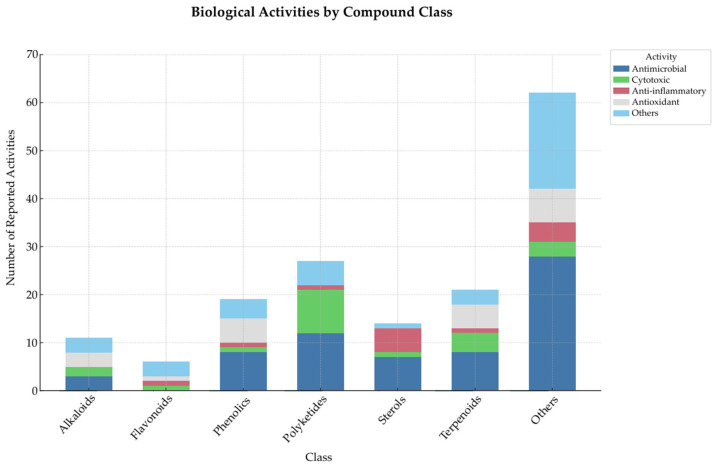
Stacked column chart depicting the distribution of major biological activities across chemical classes of secondary metabolites based on data extracted from Table 1.

Among the 111 compounds reported across 39 studies, polyketides (**24**), terpenoids (**13**), and phenolic compounds (**13**) were the most frequently identified classes of secondary metabolites produced by endophytic *Colletotrichum* species. These chemical classes also exhibited the widest range of biological activities, particularly antimicrobial and antioxidant effects. Sterols and alkaloids followed closely, while flavonoids were the least represented. Notably, many compounds exhibited multitarget activity profiles, especially within the polyketides and terpenoids classes. This distribution underscores the strong biotechnological potential of these fungi, revealing consistent correlations between chemical classes and bioactivity profiles. To better illustrate these trends, a stacked column chart was constructed (Figure 3), summarizing the distribution of compound classes in relation to their most frequently reported biological activities, based on the data presented in Table 1.

Based on the tabulated data, beyond their antimicrobial profile, polyketides also exhibited cytotoxic activity, with nine compounds reported, reinforcing their potential interest for anticancer research. Anti-inflammatory and other biological effects were less frequently reported for this class. Terpenoids were primarily associated with antimicrobial activity (five compounds), followed by examples of cytotoxic and antioxidant properties, reflecting their pharmacological diversity. Phenolic compounds showed a predominant antioxidant profile with five compounds, alongside occasional reports of antimicrobial and cytotoxic activities. Sterols were mainly linked to antimicrobial and antioxidant effects, while alkaloids were divided between antimicrobial and cytotoxic activities, both of which are relevant for anti-infective and anticancer research. Flavonoids, although the least represented, demonstrated a varied biological profile, including antimicrobial, cytotoxic, anti-inflammatory, and antioxidant activities. These data emphasize the chemical classes and bioactivities of greatest scientific interest, particularly those with potential for pharmacological development.

In addition to the predominant classes, a variety of structurally diverse compounds were identified, albeit in smaller numbers. These include metabolites such as fatty acids, macrolides, lignans, diketopiperazines, and polycyclic aromatic hydrocarbons, among others. Although less numerous, some of these compounds also exhibited biological activities that are mainly antimicrobial, with occasional reports of cytotoxic or antioxidant effects. This highlights the broad chemical and functional diversity of secondary metabolites produced by endophytic *Colletotrichum* species.

**Figure 4:**
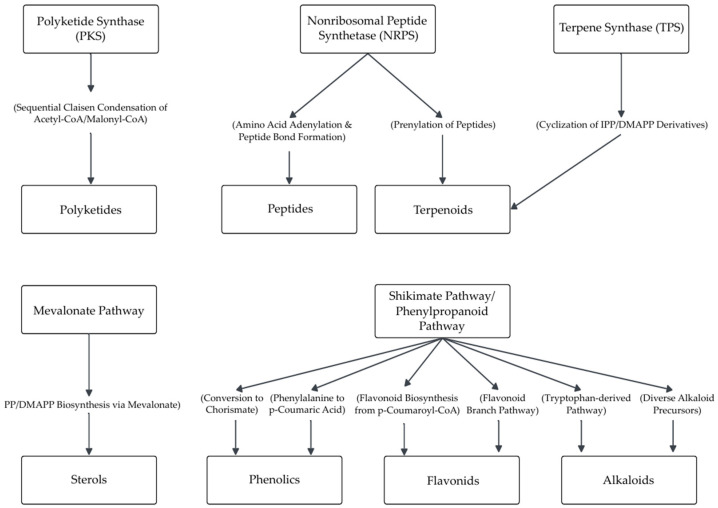
Schematic representation of the main biosynthetic pathways potentially involved in the production of major secondary metabolites identified in endophytic *Colletotrichum* species. Polyketides and peptides are synthesized via polyketide synthase (PKS) and nonribosomal peptide synthetase (NRPS) systems, respectively. Terpenoids and sterols derive from terpene synthases (TPSs) and the mevalonate pathway. Shikimate and phenylpropanoid pathways contribute to phenolics, flavonoids, and some alkaloids.

To provide a biosynthetic perspective on the chemical diversity observed, Figure 4 illustrates the main biosynthetic pathways that could potentially account for the production of the major classes of secondary metabolites identified in this review, namely, alkaloids, flavonoids, phenolics, polyketides, sterols, and terpenoids. Although the precise biosynthetic origins of all 111 compounds remain unconfirmed, these pathways, including PKS, NRPS, TPS, mevalonate, and shikimate/phenylpropanoid routes, represent plausible metabolic routes for the predominant chemical classes identified. Notable examples include ergosterol and derivatives (**4**, **5**, **9**, **11**), apigenin-8-C-*β*-D-glucopyranoside (**17**), pyrenocines (**26**, **27**, **29**–**31**), macommelin-9-acetate (**28**), piperine (**33**), 10-hydroxycamptothecin (**34**), and phenolic acids such as 2-(4-hydroxyphenyl)acetic acid (**39**) and 2-(2-hydroxyphenyl)acetic acid (**40**) [29,46,47,48,56,57,58,59]. This biosynthetic overview underscores the metabolic adaptability of *Colletotrichum* endophytes and their promise as sources of novel compounds with pharmacological potential.

## 4. Discussion

The data reviewed indicated that endophytic species of *Colletotrichum* are prolific producers of a wide array of bioactive metabolites. Among them, *C. gloeosporioides* is the most frequently investigated, and it is commonly found across a wide range of host plants and ecological niches. Its broad host adaptability likely contributes to its extensive metabolic output, which includes several compounds with potent antimicrobial, antioxidant, and cytotoxic activities [30,47]. Other species, such as *C. capsici* and *C. queenslandicum*, also contribute significantly to the diversity of *Colletotrichum*-derived natural products. However, many studies report only *Colletotrichum* sp., without species-level identification, which may be attributed to the high morphological similarity within the genus and the limited use of molecular methods. Even when molecular analyses are performed, phylogenetic resolution often reveals close genetic proximity and clustering among species, leading many identifications to remain at the genus level [29,46,50,53,57,63].

Numerous metabolites from these species have been characterized, predominantly belonging to polyketides, terpenoids, phenolic compounds, sterols, and alkaloids, with flavonoids being less common. This chemical profile reflects the genus’s biosynthetic adaptability and its relevance for pharmaceutical and agricultural applications [30,43,52,61,69]. Polyketides and terpenoids, in particular, exhibit a broad range of biological activities, including antimicrobial, antioxidant, cytotoxic, and anti-inflammatory effects. Sterols, especially ergosterol and its oxygenated derivatives, are frequently associated with anti-inflammatory properties, while tryptophan-derived compounds, such as indole-3-acetic acid (**3**), a known plant growth promoter, have been observed in species associated with medicinal plants like *C. fructicola* and *C. siamense*. Additional compounds like apigenin, piperine, and various cyclic peptides further expand the genus’s chemical diversity [29,41,42,50,58].

Available evidence suggests that specific structural features in metabolites derived from *Colletotrichum* are associated with distinct biological functions. For instance, peroxide groups in sterols are frequently linked to enhanced antifungal activity, as these moieties facilitate oxidative stress that disrupts fungal membrane integrity. Similarly, prenylated alkaloids often display intensified antimicrobial or neuroactive properties, likely due to increased lipophilicity that promotes membrane interaction and cellular uptake. Polyketides bearing aromatic rings and hydroxyl substitutions have demonstrated cytotoxic effects against tumor cell lines, potentially through mechanisms such as DNA intercalation, topoisomerase inhibition, or induction of apoptosis via oxidative pathways. These correlations highlight the relevance of structure–activity relationships (SARs), although systematic SAR studies within this genus remain scarce and fragmented. Improved characterization of these molecular features may support future strategies for compound optimization and the development of drug candidates [76,77,78].

Current biosynthetic data for endophytic *Colletotrichum* species remain limited; however, the presence of polyketides, terpenes, and alkaloids in previously reported compounds suggests the involvement of canonical biosynthetic gene clusters (BGCs), such as those encoding polyketide synthases (PKSs), nonribosomal peptide synthetases (NRPSs), and terpene synthases. Some molecules, such as morucolletotricin and isoprenylindole-3-carboxylic acid, may originate from hybrid PKS-NRPS pathways or the shikimate route, though these hypotheses lack experimental validation. Functional mapping of BGCs in endophytic *Colletotrichum* remains incomplete, yet the genus’s considerable chemical diversity reinforces the importance of integrated omics-based studies. Phylogenetic analysis of metabolite distribution has also been proposed as a strategy to identify clade-specific biosynthetic capacities, necessitating future comparative genomic investigations to elucidate the evolutionary dynamics of metabolite production [46,79,80,81].

Despite these insights, direct correlations between genes and secondary metabolites in *Colletotrichum* spp. remain poorly characterized. Evidence from related taxa using predictive genome mining suggests the presence of numerous silent or cryptic BGCs with the potential for novel chemical scaffolds. The integration of in silico pathway reconstruction with transcriptomic and metabolomic analyses is recommended for advancing the identification and functional annotation of these clusters [82]. Comparative genomic and transcriptomic studies have also provided insights into genomic plasticity and infection strategies, particularly regarding host adaptation. For example, analyses of *C. higginsianum* and *C. graminicola* reveal distinct secreted effectors and pectin-degrading enzymes, with stage-specific transcriptional shifts during infection. Comparisons between the endophytic *C. tofieldiae* and pathogenic *C. incanum* demonstrate differences in secondary metabolism gene expression and carbohydrate-active enzyme (CAZyme) repertoires, shedding light on lifestyle-related genomic traits. Although these datasets were not designed for metabolite discovery, they offer valuable frameworks for identifying candidate genes within BGCs and exploring their functional relevance [34,83,84].

This metabolic versatility is reflected in a range of biological activities observed in the isolated compounds. As illustrated in the quantitative analysis presented in this review, antimicrobial activity was the most frequently reported, followed by antioxidant, cytotoxic, and anti-inflammatory effects. For instance, compounds obtained from *C. gloeosporioides* associated with *Cymbidium aloifolium* exhibited inhibitory effects against *Escherichia coli*, *S. aureus*, and *Candida albicans*. In parallel, various unidentified *Colletotrichum* strains have been described as sources of antimicrobial, anticancer, antioxidant, cytotoxic, neuroprotective, and acetylcholinesterase-inhibitory properties [50,57,60].

In one study [29], metabolites from a *Colletotrichum* strain isolated from the stem of *Artemisia annua* included steroidal structures and three novel compounds with strong antimicrobial activity. These compounds, (**6**), (**7**), (**8**), (**10**), and (**12**), showed significant inhibition of both Gram-positive bacteria (*Bacillus subtilis*, *S. aureus*, and *Sarcina lutea*) and Gram-negative bacteria (*Pseudomonas* spp.), with minimum inhibitory concentrations (MICs) ranging from 25 to 75 µg/mL and 50–100 µg/mL against opportunistic fungi like *C. albicans* and *A. niger*. They also inhibited phytopathogens such as *Gaeumannomyces graminis*, *Rhizoctonia cerealis*, *Helminthosporium sativum*, and *Phytophthora capsici* at 200 µg/mL. Such results reinforce the genus’s biosynthetic potential and highlight medicinal plants as valuable sources of endophytic fungi with promising biotechnological applications [28,45,48,63,65,73]. In comparison, tropicicolide (**73**), isolated from *C. tropicicola*, exhibited IC_50_ values of 1.8 µg/mL against *A. fumigatus* and 7.1 µg/mL against *C. albicans*, which are substantially lower than the MICs recorded for the steroidal derivatives from the fungus isolated from *A. annua* [71].

Other species, such as *C. crassipes* isolated from *Casearia sylvestris*, have been reported to produce Cyclo-(D)-Pro-(D)-Phe (**59**) and N-(2-phenylethyl)acetamide (**60**), compounds with distinct bioactivities. N-(2-phenylethyl)acetamide demonstrated potent antifungal activity at 50 µg/mL against *Cladosporium cladosporioides* and moderate activity against *C. sphaerospermum* when tested using the thin-layer chromatography (TLC) diffusion method. Cyclo-(D)-Pro-(D)-Phe exhibited antioxidant capacity at 1 mg/mL, with structure–activity relationship analysis indicating that the presence of electron-donating groups enhances the efficiency of DPPH radical reduction, supporting the idea that the antifungal efficacy of *Colletotrichum*-derived metabolites can vary markedly across species and chemical classes [67].

*C. gloeosporioides* and *C. dematium* have also been identified in the literature as taxol producers, a compound of high relevance in anticancer therapy [54,55]. Similarly, studies on *C. acutatum* from *Angelica sinensis* described the production of C-HMMP, a multifunctional molecule with antimicrobial, antibiofilm, antioxidant, antimalarial, antiproliferative, antimutagenic, and antidiabetic activities [70]. *C. taiwanense*, *C. alatae*, and *C. brevisporum* have also been associated with antioxidant and antimicrobial properties. Collectively, the findings consolidate the role of *Colletotrichum* species as prolific sources of structurally diverse metabolites, for which their bioactivities are modulated by specific molecular frameworks [72,74,75].

Despite promising in vitro outcomes, few studies assess pharmacokinetic parameters, in vivo efficacy, or toxicity of these metabolites. Most reports are confined to screening assays using crude extracts or purified compounds at the cellular level, leaving the translational potential unclear. Evaluating metabolic stability, bioavailability, and off-target effects is essential for pharmaceutical development. Additionally, the ecological duality of the *Colletotrichum* genus, encompassing both endophytic and phytopathogenic species, raises further safety considerations, particularly for applications involving live cultures or minimally processed extracts. Clarifying the genetic and environmental triggers that mediate the shift between mutualism and pathogenicity is critical to ensure safe and reliable biotechnological applications [9,85].

Beyond safety concerns, poor reproducibility and lack of methodological standardization across studies significantly hinder the biotechnological exploitation of *Colletotrichum* spp. Variations in culture conditions, extraction protocols, and host plant associations directly affect metabolite profiles and yields, with considerable fluctuations even among strains of the same species. This scenario is further complicated by the structural complexity, chemical instability, and labor-intensive purification processes required for crude extracts, all of which hinder scalability and industrial application. Additionally, low yields (often <1 mg/mL), long fermentation periods (typically 14–30 days), and variability in biomass and metabolite production influenced by culture media composition and environmental parameters further compromise process consistency. To overcome these limitations, strategies such as culture medium optimization, co-culture systems to activate silent biosynthetic pathways, the use of bioreactors, and metabolic engineering have been proposed; however, their specific application to *Colletotrichum* still requires further investigation through targeted pilot-scale studies [86,87,88].

Although not included in the core analysis of this review, several other studies, even without compound isolation, have reported relevant bioactivities from crude extracts. These references are provided solely to reinforce the biotechnological relevance of the genus *Colletotrichum*. For instance, *C. lindemuthianum* ethyl acetate extracts showed broad-spectrum antimicrobial activity using the well diffusion method. Inhibition zones were reported as 8 mm against *S. aureus*, 6 mm against *Proteus vulgaris*, and 4 mm against *K. pneumoniae* [89]. Similarly, crude extracts of *Colletotrichum* sp. isolated from *Rauvolfia serpentina* demonstrated antibacterial activity against *E. coli* and *S. aureus*, with inhibition zones of 16 mm and 14 mm, respectively, indicating greater antibacterial activity than *C. lindemuthianum* and emphasizing species-specific differences in potency [90].

In previous studies, extracts from *C. siamense*, *C. jiangxiense*, and *C. karstii* exhibited moderate antibacterial activity, with MICs ranging from 500 to 1000 μg/mL against both Gram-positive and Gram-negative bacteria. Notably, activity was observed against clinically relevant Gram-negative strains such as *K. pneumoniae*, *S. enteritidis*, and *S. flexneri*, which are often associated with multidrug resistance. Although the MICs were relatively high, these findings highlight the potential of *Colletotrichum* species as sources of metabolites with activity against hard-to-treat Gram-negative pathogens [91]. In the context of anticancer and antioxidant potential, the ethyl acetate extract of *C. gloeosporioides* exhibited cytotoxic effects against the HCT116 and HeLa cancer cell lines, with IC_50_ values of 76.6 and 176.2 μg/mL, respectively, while also showing strong antioxidant activity against DPPH radicals (EC_50_ = 22.2 μg/mL). This combination of bioactivities is particularly relevant considering the role of oxidative stress in cancer development and progression [92].

Additional reports, such as that of Subbulakshmi et al. [93], have described that methanol extracts of *C. gloeosporioides* demonstrated antimicrobial activity against key pathogens such as *S. aureus* (12 mm), *E. coli* (12 mm), and *C. albicans* (22 mm). A notable exception in terms of potency is the work of Bin et al. [94], who explored an endophytic *Colletotrichum* species isolated from *Aegiceras corniculatum.* The ethyl acetate extract exhibited potent antibacterial activity against multidrug-resistant pathogens, with MICs of 4 μg/mL against *K. pneumoniae* and 0.5 μg/mL against *Acinetobacter baumannii*, indicating a noteworthy therapeutic potential even prior to compound purification.

Collectively, these studies indicate that the bioactivity of *Colletotrichum* crude extracts varies depending on species identity, host plant origins, and the solvent used for extraction. The highest antibacterial potency was observed in the ethyl acetate extract from the mangrove-derived *Colletotrichum* sp., which inhibited multidrug-resistant bacteria, while terrestrial species displayed broader but less potent antimicrobial spectra. Compared to purified compounds, crude extracts are valuable for initial screenings but are limited by their complex composition, which hinders precise attribution of bioactivity. Isolated compounds, in turn, offer higher potency and allow the identification of active chemical classes, enabling deeper pharmacological insights. Progress in harnessing the therapeutic potential of *Colletotrichum* requires bioassay-guided fractionation, structural characterization, and assessments of species and ecological diversity to better link metabolites to bioactivities [91,92,93,94].

Despite their biosynthetic potential, real-world applications of *Colletotrichum*-derived compounds face industrial and regulatory challenges, including fermentation scalability, batch consistency, regulatory approval, and environmental impacts. Addressing such barriers early in the discovery process is critical for commercial feasibility [95]. Omics-based tools, particularly genomics and metabolomics, are essential to unravel *Colletotrichum*’s metabolic complexity. Advances in genome sequencing and transcriptomics reveal gene expression patterns under different hosts or environments. These tools also facilitate studies on metabolic host–endophyte interplay, co-metabolite production, and biosynthetic regulation, which are pivotal for enhancing compound discovery [96].

Studies have demonstrated that some metabolites produced by endophytic fungi may arise from co-metabolic pathways or are influenced by host-derived compounds. For instance, precursors or inducers present in host plant tissues can activate otherwise silent biosynthetic pathways in the fungus, resulting in the production of unique metabolites. Certain biosynthetic routes are expressed only under symbiotic conditions or in response to plant stress signals, reflecting functional co-evolution. In planta-based approaches, comparative metabolomics, and co-culture experiments could uncover biosynthetic potentials that are not observable under axenic culture conditions [96,97].

In this context, co-culture strategies, whether involving host plants, other microorganisms, or even competing fungi, have been increasingly recognized as effective tools for eliciting the expression of cryptic or silent biosynthetic gene clusters (BGCs). By simulating natural ecological interactions, these approaches can induce metabolic exchanges or competitive responses that activate novel biosynthetic pathways that are otherwise dormant in monocultures. This approach could be particularly valuable for *Colletotrichum*, as genomic analyses have identified numerous biosynthetic gene clusters for which their full expression profiles under different culture conditions remain largely unexplored. Additionally, the integration of multi-omics approaches, combining genomics, transcriptomics, and metabolomics, provides a comprehensive framework to map and link these BGCs to specific chemical outputs. Such strategies not only aid in deciphering the molecular basis of metabolite production but also facilitate the discovery of novel compounds with high biotechnological potential [79,96,97].

The presence of oxygenated sterols, flavonoids, terpenoids, and alkaloids in these endophytic fungi aligns with the genus’s chemical profile, further substantiating its biosynthetic richness and adaptive metabolic capacity. These findings emphasize the importance of expanding research on endophytic *Colletotrichum* species, particularly through the integration of advanced isolation techniques, structural elucidation, and multi-target biological screening. Such efforts are essential to fully harness their chemical potential and to uncover novel compounds with applications in the pharmaceutical, agricultural, and industrial sectors [98].

## 5. Conclusions

This review highlights that the endophytic species of the genus *Colletotrichum* represent a promising source of bioactive metabolites with a wide spectrum of biological activities. The compounds identified to date have demonstrated considerable potential for applications in the pharmaceutical, agricultural, and industrial sectors, largely due to their structural and functional diversity. Importantly, even in the absence of isolated pure compounds, crude extracts from these fungi frequently exhibit significant bioactivity, suggesting possible synergistic interactions among secondary metabolites.

Despite this promise, these fungi remain underexplored, particularly in tropical ecosystems. Expanding research efforts on their ecological diversity, strain isolation, and comprehensive metabolite profiling is crucial to unlock their potential. Future research should prioritize the following concrete steps: (i) systematic bioprospecting in under-sampled tropical and subtropical regions; (ii) rigorous dereplication and identification of novel metabolites through integrated omics (genomics, metabolomics, and transcriptomics); and (iii) development of strain libraries with taxonomic validation and biosynthetic potential assessments.

Additionally, researchers should aim to (iv) characterize biosynthetic gene clusters (BGCs) through genome mining and expression studies; (v) investigate structure–activity relationships (SARs) using targeted synthetic modifications, cheminformatics, and molecular modeling; and (vi) evaluate bioactive candidates in validated in vivo models to assess pharmacokinetics, toxicity, and efficacy.

Moreover, the establishment of sustainable and scalable production methods will be essential for the industrial application of these metabolites. Optimizing bioprocesses, including culture media, fermentation parameters, co-cultivation strategies, and bioreactor configurations, can significantly enhance yield and cost-effectiveness. Functional validation through in vitro and in vivo assays is also necessary to confirm the therapeutic or agricultural efficacy of promising metabolites. Fostering interdisciplinary collaboration across microbiology, natural product chemistry, pharmacology, and biotechnology will be fundamental to translating these discoveries into real-world solutions.

Overall, advancing research on endophytic *Colletotrichum* species could contribute significantly to the discovery of novel natural products and sustainable solutions for crop protection and health care. This aligns with the broader goals in global health, sustainable agriculture, and biodiversity conservation, reinforcing the value of this fungal group in addressing contemporary scientific and societal challenges. Importantly, the ecological duality of the *Colletotrichum* genus, encompassing both endophytic and phytopathogenic species, should be carefully considered in future studies to ensure the safe and responsible development of these applications. Understanding the factors that regulate this dual behavior may enhance the biotechnological exploitation of these fungi while minimizing potential risks.

## Figures and Tables

**Table 1:** Bioactive compounds identified from endophytic *Colletotrichum* species.

Endophytic Fungus	Host Plant(s)	Identified Compound(s)	Chemical Class	Biological Activity	References
*C. fragariae*	*Fragaria* spp.	Colletofragarones; A1 (**1**) e A2 (**2**)	Polyketides	Self-germination inhibitory activity	[40]
*Colletotrichum* sp. (unresolved taxon); *C. gloeosporioides*; *C. fructicola*; *C. siamense*; *C. queenslandicum*	*Artemisia annua*; *Piper nigrum*; *Vincetoxicum hirsutum*; *Cymbidium aloifolium*; *Vernonia amygdalina*; *Morus australis*	Indole-3-acetic acid (IAA) * (**3**)	Indole alkaloid	Plant growth-promoting activity	[29,41,42,43,44,45,46]
*Colletotrichum* sp. (unresolved taxon); *C. gloeosporioides*; *C. queenslandicum*	*Artemisia annua*; *Virola Michelii*; *Uncaria rhynchophylla*; *Morus australis*	Ergosterol * (**4**)	Sterol	Anti-inflammatory activity; cytotoxic activity	[29,46,47,48]
*Colletotrichum* sp. (unresolved taxon); *C. gloeosporioides*	*Artemisia annua*; *Virola michelli*; *Uncaria rhynchophylla*	Ergosterol peroxide * (**5**)	Sterol	Anti-inflammatory activity; PI3K*α* inhibitory activity	[29,47,48]
*Colletotrichum* sp. (unresolved taxon)	*Artemisia annua*	Isoprenylindole-3-carboxylic acid (**6**); 3,5-Dihydroxy-6-acetoxyergosta-7,22-dienoic acid (**7**); 3,5-Dihydroxy-6-phenylacetoxyergosta-7,22-dienoic acid (**8**); 3*β*,5*α*,6*β*-Trihydroxyergosta-7,22-diene (**9**); 3-Oxoergosta-4,6,8(14),22-tetraene (**10**); 3*β*-Hydroxy-5*α*,8*α*-epidioxyergosta-6,9(11),22-triene (**11**); 3*β*-Hydroxyergosta-5-ene (**12**); 3-Oxoergosta-4-ene (**13**)	Indole alkaloid; Sterol derivative; Sterol derivative; Sterol derivative; Sterol derivative; Sterol derivative; Sterol; Sterol derivative	Antimicrobial activity	[29]
*C. gloeosporioides*	*Vincetoxicum hirsutum*	Coletotric acid (**14**)	Polyketide	PTP1B inhibitory activity; anti-inflammatory activity	[43]
*C. gloeosporioides*; *C. acutatum*	*Cryptocaryamandioccana*; *Fragaria × ananassa*	(4R)-4,8-Dihydroxy-*α*-tetralone * (**15**); cis-4-Hydroxy-6-deoxyscytalone * (**16**)	Polyketides	Antifungal activity	[30,49]
*Colletotrichum* sp. (unresolved taxon) (NTB-2)	*Ginkgo biloba*	Apigenin-8-*C*-*β*-D-glucopyranoside (**17**)	Flavonoid glycoside	Anti-inflammatory activity; antioxidant activity; antihypertensive activity; antihepatotoxic activity; Antiarteriosclerotic activity	[50]
*C. gloeosporioides*	*Forsythia suspensa*	Phillyrin (**18**)	Lignan glycoside	Antioxidant activity; anti-inflammatory activity; antipyretic activity	[51]
*C. gloeosporioides*	*Phlogacanthus thyrsiflorus*	2,4-Bis(1,1-dimethylethyl)phenol (**19**); Hexadecanoic acid (**20**); Methyl octadecanoate (**21**)	Phenolic compound; Fatty acid; Fatty acid ester	Antimicrobial activity; antioxidant activity	[52]
*Colletotrichum* sp. (unresolved taxon); *C. gloeosporioides*	*Pandanus amaryllifolius*	Colletotriolide (**22**); Tyrosol C (**23**)	Macrolide; Phenolic compound	Antibacterial activity	[53]
*C. gloeosporioides C. dematium* CBP2;	*Tectona grandis*; Not specified (KACC)	Taxol (Paclitaxel) * (**24**)	Diterpenoid	Cytotoxic activity	[54,55]
*C. siamense*; *C. gloeosporioides*	*Piper nigrum*; *Magnolia champaca*	Uracil * (**25**)	Pyrimidine derivative	Acetylcholinesterase (AChE) inhibitory activity; antifungal activity	[41,56]
*Colletotrichum* sp. (unresolved taxon) (HCCB03289)	*Ludwigia prostrata*	Pyrenocine N (**26**); Pyrenocine O (**27**); Macommelin-9-acetate (**28**); Pyrenocine A (**29**); Pyrenocine B (**30**); Pyrenocine E (**31**); Novaezelandine A (**32**)	Polyketide; Polyketide; Diterpenoid; Polyketide; Polyketide; Polyketide; Sesquiterpenoid	Cytotoxic activity	[57]
*C. gloeosporioides*	*Piper nigrum*	Piperine (**33**)	Alkaloid	Antimicrobial activity; antioxidant activity; cytotoxic activity	[58]
*C. gloeosporioides* XSXY05	*Camptotheca acuminata*	10-Hydroxycamptothecin (**34**)	Alkaloid	Cytotoxic activity	[59]
*C. gloeosporioides*	*Magnolia champaca*	2-Phenylethyl 1H-indol-3-ylacetate (**35**); Cyclo-(S-Pro-S-Tyr) (**36**); Cyclo-(S-Pro-S-Val) (**37**); 2-(2-Aminophenyl)acetic acid (**38**); 2-(4-Hydroxyphenyl)acetic acid (**39**); 2-(2-Hydroxyphenyl)acetic acid (**40**); 4-Hydroxybenzamide (**41**)	Indole derivative; Diketopiperazines; Aromatic amino acid derivative; Phenolic acid; Phenolic acid; Benzamide derivative;	Antifungal activity; acetylcholinesterase (AChE) inhibitory activity	[56]
*Colletotrichum* sp. (unresolved taxon)	*Huperzia serrata*	Huperzine A (**42**)	Alkaloid	Antioxidant activity; acetylcholinesterase (AChE) inhibitory activity	[60]
*C. gloeosporioides*	*Lannea corammendalica*	9-Octadecenamide (**43**); Hexadecenamide (**44**); Diethyl phthalate (**45**)	Fatty acid amide; Fatty acid amide; Phthalate ester	Antimicrobial activity	[61]
*Colletotrichum* sp. (unresolved taxon) (BS4)	*Buxus sinica*	Colletotrichone A (**46**); Colletotrichone B (**47**); Colletotrichone C (**48**); Chermesinone B (**49**)	Polyketides	Antibacterial activity; cytotoxic activity	[62]
*C. capsici*	*Siegesbeckia pubescens*	Citrinal A (**50**); Citrinal B (**51**)	Polyketides	Cytotoxic activity	[63]
*C. gloeosporioides*	*Virola michelii*	*β*-Sitosterol; (**52**); Stigmasterol; (**53**); Sitostenone (**54**)	Sterol; Sterol; Sterol derivative	Anti-inflammatory activity	[47]
*C. gloeosporioides*	*Cymbidium aloifolium*; *Virola michelii*	Squalene (**55**)	Triterpenoid	Antimicrobial activity; antioxidant activity; cytotoxic activity	[44,47]
*C. capsici* KT37396; *C. taiwanense PI-3* KX580307	*Passiflora incarnata*	Chrysin (**56**)	Flavonoid	Cytotoxic activity	[64]
*Colletotrichum* sp. (unresolved taxon) (JS-0367)	*Morus alba*	Evariquinone (**57**)	Anthraquinone	Neuroprotective activity	[65]
*C. gloeosporioides*	*Centella asiatica*	Asiaticoside (**58**)	Triterpenoid glycoside	Immunomodulatory activity; antidepressant activity;	[66]
*C. crassipes*	*Casearia sylvestris*	Cyclo-(D-Pro-D-Phe) (**59**); N-(2-Phenylethyl) acetamide; (**60**)	Diketopiperazine; Aromatic amide	Antioxidant activity; antifungal activity	[67]
*C. gloeosporioides* GT-7	*Uncaria rhynchophylla*	Cyclo-(L-Leu-L-Leu) (**61**); Brevianamide F (**62**)	Diketopiperazine; Indole alkaloid	PI3K*α* inhibitory activity	[48]
*C. gloeosporioides* B12	*Illigera rhodantha*	Colletolides A (**63**) e B (**64**)	Polyketides	Antibacterial activity	[68]
*C. gloeosporioides*	*Carica papaya*	Aureonitol (**65**); Protocatechuic acid (**66**); Glucobrassicin (**67**)	Lignan; Phenolic acid; Indole glucosinolate	Antiviral activity; antibacterial activity; cytotoxic activity	[69]
*C. gloeosporioides*	*Vincetoxicum hirsutum*	Lumichrome (**68**); *β*-Acetyltryptamine (**69**); Cyclo-(Trp-Phe) (**70**); (Z)-2-(2-(2-(4-hydroxyphenyl)acetoxy)ethyl)but-2-enoic acid (**71**);	Flavin derivative; Indole derivative; Diketopiperazine; Phenolic acid derivative	PTP1B inhibitory activity; anti-inflammatory activity	[43]
*C. acutatum*	*Angelica sinensis*	5-(1-Hydroxybutyl)-4-methoxy-3-methyl-2H-pyran-2-one (C-HMMP) (**72**)	Pyrone derivative	Antimicrobial activity; antibiofilm activity; antioxidant activity; antimalarial activity; antiproliferative activity	[70]
*C. tropicicola* F10154	Native plants from Singapore (not specified)	Tropicicolide (**73**)	Polyketide	Antifungal activity	[71]
*Colletotrichum* sp. (unresolved taxon) (AP-4)	*Andrographis paniculata*	Andrographolide (AD) (**74**); Neandrographolide (NAD) (**75**); 14-Deoxyandrographolide (DAD) (**76**); 14-Deoxy-11,12-didehydroandrographolide (DDAD) (**77**)	Diterpenoids	Antioxidant activity; antibacterial activity	[28]
*C. alatae* LCS1	*Lycopodium clavatum*	Bisabolol (**78**); Oxalic acid (**79**); 7-Isopropyl-1-methylphenanthrene (**80**); Pterine-6-carboxylic acid (**81**); Dimethylamine (**82**); 2-(2-Aminopropyl)phenol (**83**); Phthalic acid (**84**); Naphthalene (**85**)	Sesquiterpenoid; Dicarboxylic acid; Polycyclic aromatic hydrocarbon; Pteridine derivative; Amine; Aromatic amine derivative; Dicarboxylic acid; Polycyclic aromatic hydrocarbon	Antibacterial activity; antioxidant activity	[72]
*Colletotrichum* sp. (unresolved taxon)	*Vernonia amygdalina*	Palitantin (**86**); Cladosporin (**87**); p-Hydroxybenzaldehyde (**88**); Desmethyldichloro-diaportin (**89**); p-Hydroxybenzoic acid (**90**)	Alkaloid; Polyketide; Phenolic aldehyde; Polyketide; Phenolic acid;	Antimicrobial activity; antioxidant activity	[73]
*C. gloeosporioides*	*Cymbidium aloifolium*	Farnesol (**91**); Tryptophan (**92**); 4-Hydroxybenzyl alcohol (**93**)	Sesquiterpenoid alcohol; Aromatic amino acid; Phenolic alcohol	Antimicrobial activity	[44]
*C. brevisporum* JPSK19	*Bergenia ciliata*	4-(1,1-Dimethylpropyl)phenol (**94**); 1-Docosene (**95**)	Phenolic compound; Alkene	Antibacterial activity; antioxidant activity	[74]
*Colletotrichum* sp. (unresolved taxon)	*Vernonia amygdalina*	Acropyrone (**96**); Beauvericin (**97**); Indole-3-carbaldehyde (**98**); Rocaglamide A (**99**)	Polyketide; Cyclic hexadepsipeptide; Indole derivative; Flavagline	Antimicrobial activity;	[45]
*C. taiwanense* BPSRJ3	*Vanda cristata*	Cyclobarbital (**100**); Phenanthrene (**101**); 2,6-Di-tert-butylphenol (**102**); 3-Carene (**103**); Camphene (**104**); 1-Fluorododecane (**105**); 17-Octadecenoic acid (**106**); 2,6-Dihydroxyacetophenone (**107**)	Barbiturate; Polycyclic aromatic hydrocarbon; Phenolic compound; Monoterpene; Monoterpene; Fluoroalkane; Unsaturated fatty acid; Phenolic ketone;	Antioxidant activity; antimicrobial activity; anti-inflammatory activity; cytotoxic activity	[75]
*C. queenslandicum*	*Morus australis*	Morucolletotricin (**108**); Tryptophol (**109**); Phomopyronol (**110**); 2-(3-Aminophenyl)acetic acid (**111**)	Polyketide; Indole derivative; Polyketide; Aromatic amino acid derivative	Cytotoxic activity	[46]

* The same compound was identified from multiple *Colletotrichum* species associated with distinct host plants, as consolidated in this table.

## Data Availability

No new data were created or analyzed in this study. Data sharing is not applicable to this article.
